# Stroke Status Evoked Adhesion Molecule Genetic Alterations in Astrocytes Isolated from Stroke-Prone Spontaneously Hypertensive Rats and the Apigenin Inhibition of Their Expression

**DOI:** 10.4061/2010/386389

**Published:** 2009-12-20

**Authors:** Kazuo Yamagata, Takuya Kitazawa, Masahiro Shinoda, Chika Tagawa, Makoto Chino, Hiroshi Matsufuji

**Affiliations:** Department of Food Science and Technology, College of Bioresource Sciences, Nihon University (NUBS), Kanagawa 252-8510, Japan

## Abstract

We examined the possibility that the expression of adhesion molecules is regulated differently in cultured astrocytes from stroke-prone spontaneously hypertensive rats (SHRSP/IZM) rats than in those from Wistar Kyoto rats (WKY/IZM) by tumor necrosis factor-alpha (TNF-*α*) or hypoxia and reoxygenation (H/R) and the inhibitory effects of apigenin. It was found that the expression of vascular cell adhesion molecule-1 (VCAM-1) by TNF-*α* in astrocytes isolated from SHRSP/IZM was increased compared with that in WKY/IZM. The expression of monocyte chemotactic protein-1 (MCP-1) mRNA induced by H/R in SHRSP/IZM astrocytes was increased compared with that in normal oxygen concentrations. Apigenin strongly attenuated TNF-*α*-induced VCAM-1 mRNA and protein expression and suppressed the adhesion of U937 cells and SHRSP/IZM astrocytes. These results suggest that the expression levels of adhesion molecules during H/R affect disease outcome and can drive SHRSP/IZM to stroke. It is suggested that apigenin regulates adhesion molecule expression in reactive astrocytes during ischemia.

## 1. Introduction

The onset of cerebral ischemia triggers a cascade of proinflammatory molecular and cellular events. An important role has been indicated for adhesion molecules such as intercellular adhesion molecule-1 (ICAM-1) and p-selectin in the recruitment of leukocytes in the postischemic cerebral microvasculature. Blockade or genetic deletion of these adhesion molecules has been demonstrated to reduce infarct volume, edema, and/or mortality in different animal models of ischemic stroke [1]. Inflammatory cytokines such as tumor necrosis factor-alpha (TNF-*α*) and interleukin-6 (IL-6) and several adhesion molecules are related to the presence of early neurological deterioration and infarct volume [2, 3]. In particular, TNF-*α* and IL-6 are related to the risk of recurrent ischemic stroke independently of conventional risk markers [4, 5]. TNF-*α* is produced by microglial cells and infiltrating macrophages following ischemic stroke and may have neurotoxic and/or neuroprotective effects [6]. Predictors of adverse stroke outcome include TNF-*α*, ICAM-1, and IL-6 [7]. 

The stroke-prone spontaneously hypertensive rat (SHRSP/IZM) is characterized by cerebral hemorrhaging and infarction and is extensively used for studying the effects of drugs on stroke [8]. In other words, SHRSP/IZM is regarded as a useful genetic model of human hypertensive stroke [9, 10]. Cerebral ischemia in SHRSP/IZM induces a massive efflux of glutamate, causing delayed neuronal death in the CA 1 region of the hippocampus, whereas Wister Kyoto rats (WKY/IZM) lack these characteristics under the same conditions [11]. The hippocampal neurons in SHRSP/IZM are innately vulnerable to ischemia, but Ca^2+^ channel blockers prevent neuronal cell death in SHRSP [12]. SHRSP/IZM neurons are more vulnerable than WKY/IZM neurons during hypoxia and reoxygenation (H/R) [13–15]. The expression of monocyte chemoattractant protein-1 (MCP-1) was investigated in SHRSP/IZM rats with transient ischemia in order to study the involvement of the infiltration of monocytes from the blood in the mechanism of ischemia-related neuronal death [16]. After ischemia and reperfusion, MCP-1 mRNA was clearly induced in CA1, CA4, and the molecular layer of the dentate gyrus. The majority of MCP-1 expression occurred in astrocytes. These findings suggest that astrocytes that express MCP-1 are related to the pathological events of delayed neuronal death in SHRSP/IZM [16]. It has been reported that the expression of adhesion molecules in the astrocytes of SHRSP/IZM is closely related to ischemia/reperfusion-induced neuronal damage; however, the precise regulation of the expression of adhesion molecules in SHRSP/IZM astrocytes has not been elucidated. 

Dietary flavonoids act as antioxidants in human plasma and other extracellular fluids and protect low-density lipoproteins (LDL) from oxidation [17]. The beneficial effects of dietary flavonoids can be explained by their antioxidative capacity and subsequent modulation of intercellular redox maintenance, cell signaling, and gene expression [17]. Moreover, they reduce the cytokine-induced expression of e-selection, ICAM-1, and vascular cell adhesion molecule-1 (VCAM-1) in umbilical vein endothelial cells [18]. Furthermore, in rat brain astrocytes, the activity and mRNA of superoxide dismutase (SOD) were markedly increased after incubation with catechin at a low concentration for 2 days in culture [19]. On the other hand, resveratrol significantly attenuates the TNF-*α* induced expression of nuclear factor-kappa B (NF-kappa B)-dependent inflammatory markers, inducible nitric oxide synthase (iNOS), IL-6, bone morphogenetic protein-2 (BMP-2), ICAM-1, and VCAM-1 [20]. In addition, a recent study demonstrated the protective effects of resveratrol on enhanced hydrogen peroxide toxicity in primary astrocytes [21]. Also, apigenin effectively reduces TNF-*α* induced ICAM-1 upregulation in vivo through a mechanism that is unrelated to free radical scavenging [22]. In this study, we compared the drugs N-acetylcysteine (NAC), ebeselen, and pioglitazone to determine the effects of apigenin and resveratrol. It is recognized that apigenin and resveratrol decrease expressions of iNOS or VCAM-1 through inhibition of NF-kappa B [20], while ebeselen, a therapeutic drug for stroke, attenuates TNF*α*-induced ICAM-1 and VCAM-1 by inhibition of NF-kappa B. Furthermore, pioglitazone, a therapeutic drug for diabetes, reduces MCP-1 by stimulation of PPAR-*γ* [23], and NAC has an antioxidant effect and an inhibitory effect of NF-kappa B. 

 Astrocyte activation during H/R has been implicated in the pathogenesis of SHRSP/IZM [24–26]. However, the ischemic changes in astrocyte adhesion molecules of SHRSP/IZM have not been clarified. On the other hand, TNF-*α* is produced by brain cells after various stimuli such as ischemia. TNF-*α* induces the expression of adhesion molecules for leukocytes in astrocytes. The expression of adhesion molecules in the astrocytes by TNF-*α* is closely related to ischemic stroke development in the acute setting. Therefore, we were interested the expression of ICAM-1, MCP-1, and VCAM-1 in the astrocytes of SHRSP/IZM rats during stroke statuses such as H/R and TNF-*α* stimulation. In this study, we investigated the expression of ICAM-1, MCP-1, and VCAM-1 during H/R in cultured astrocytes isolated from SHRSP/IZM. In addition, we were interested in whether there are any modifying effects on H/R-induced expression in astrocytes of SHRSP/IZM rats caused by apigenin, resveratrol, or other reagents such as pioglitazone. In other words, we examined whether flavonoids can modulate gene expression.

## 2. Materials and Methods

### 2.1. Materials

Recombinant human TNF-*α* was purchased from Roche Applied Science (Roche Applied Science, Indianapolis, IN). Apigenin, resveratrol, ebselen, and N-acetylcysteine (NAC) were obtained from Sigma-Aldrich, Inc. (St. Louis, MO). Pioglitazone was obtained from Takeda Pharmaceutical (Osaka, Japan).

### 2.2. Cell Culture

Primary astrocytes were prepared from the cerebral cortices of fetal WKY/IZM and SHRSP/IZM rats as described previously [27]. These primary astrocytes were maintained in Dulbecco's modified Eagle's medium (DMEM) supplemented with 10% fetal bovine serum (FBS, Gibco Invitrogen, Grand Island, NY) at 37°C in a CO_2_ incubator. The astrocytes were separated from other cells by shaking the culture flask at 220 rpm for 5 hours. Cultures with a homogeneous cell population (consisting of >95% astrocytes as determined by glial fibrillary acidic protein staining) were used for the experiments [28]. Astrocytes were inoculated on culture plates (Corning, Vernon, NY, USA) and cultivated in DMEM containing 10% FBS until confluence was reached. 

Human monocytic U937 cells were purchased from the American Type Culture Collection (Rockville, MD, USA). They were maintained in RPM-1640 medium supplemented with 10% FBS. U937 cells were grown in suspension cultures and subcultured 1 : 4 three times per week in 75-cm^2^ culture flasks.

### 2.3. Treatment of Cultures

The astrocytes were plated on 100 mm cell culture dishes (Sumitomo Bakelite Co., LTD, Tokyo, Japan) or 24-well plates (Corning) at an initial density of 15 × 10^4^ cells per cm^2^. The cells were grown at 37°C, under 5% CO_2_, in a humidified atmosphere until confluent, which typically took 24–48 hours. They were then incubated with or without TNF-*α* (10 ng/mL) and different concentrations of apigenin or resveratrol (10, 30, or 50 *μ*M) for 0–24 hours.

### 2.4. Hypoxia and Reoxygenation

The astrocytes were incubated in 1% O_2_, 94% N_2_, and 5% CO_2_ (hypoxia) for 24 hours. After hypoxic culture, they were maintained in air (21% O_2_) and 5% CO_2_ (reoxygenation) for 3 hours as previously described [13, 15]. Some groups underwent further incubation (some groups before hypoxia and others after hypoxia) and received different concentrations of apigenin, resveratrol, ebselen, NAC, or pioglitazone.

### 2.5. Total RNA Extraction and cDNA

 Total RNA was extracted from cultured neurons using Trizol reagent (Gibco Invitrogen). DNase I (Gibco, Invitrogen) was used to treat RNA samples at room temperature for 15 minutes to remove genomic DNA. DNase I was heated for 15 minutes at 65°C to inactivate it. First-strand cDNA synthesis was performed with Superscript II (Gibco, Invitrogen).

### 2.6. RT-PCR

 PCR was performed for adhesion molecule gene expression. Primers were selected from Genbank and designated using the primer design software Primer Express (Applied Biosystems). Table 1 summarizes the primer sets used. The reaction mixture (50 *μ*L) contained 200 ng of the cDNA sample; 1.25 U of Ampli-Taq DNA polymerase; 1× PCR reaction buffer; 200 mM of each primer; 200 mM of dATP, dCTP, dGTP, and dTTP; and 1.5 mM MgCl_2_ (Applied Biosystems). The thermal cycling conditions were 5 minutes at 95°C, then 35 cycles of 94°C for 30 seconds, 55°C for 30 seconds, and 72°C for 30 seconds. After amplification, 10 *μ*L of the reaction mixture were electrophoresed on a 2% NuSive : agarose (3 : 1) (FMC product, Rockland, ME) gel and visualized with UV illumination after staining with ethidium bromide. The amount of mRNA expressed was measured relative to that per 18s ribosomal RNA. The PCR conditions were confirmed to be as described in the abovementioned report and met the standard composition and conditions used.

### 2.7. Western Blot Analysis

 The cells were lysed in RIPA buffer (50 mM Tris-HCl, pH 8.0, 150 mM NaCl, 1% Nonidet P-40, 0.5% deoxycholic acid, 0.1% SDS), and the cell lysates (30–50 *μ*g protein) were separated by SDS-PAGE and transferred to polyvinylidene difluoride membranes. The membranes were incubated with antibody against the target protein for 2 hours. After being washed twice, the membranes were incubated with a horseradish peroxidase-conjugated secondary antibody, and the protein levels were detected by the enhanced chemiluminescence system (Invitrogen).

### 2.8. Astrocyte-U937 Cell Adhesion Assay

For the adhesion assay, SHRSP/IZM astrocytes were resuspended in DMEM medium with 10% FBS at the desired density and plated in either 24-well or 96-well plates. Human monocyte-like U937 cells (1 × 10^6^ cells/mL) were added and incubated with astrocytes at 37°C for 3 hours. At the end of the incubation, the wells were filled with culture medium and aspirated three times to remove unbound U937 cells. The adhesion of U937 cells to the astrocytes was measured using quantitative monolayer adhesion assays, similar to those previously described elsewhere [29]. Briefly, the number of bound U937 cells per square millimeter was determined by direct microscopy. One randomly selected central field and four peripheral fields of the intact endothelial monolayers were examined with an ocular grid using phase-contrast microscopy (×200). The total number of bound U937 cells was determined by a hemacytometer. The residual U937 cells that were not removed from the empty wells by the washing procedure typically represented −2% of the bound U937 cells in each experiment.

### 2.9. Statistical Analysis

Data are presented as means ± SD. The significance of differences was determined using Fisher's protected least significant difference (PLSD) method following an analysis of variance (ANOVA). 

## 3. Results

### 3.1. A Comparison between the Gene Expression Levels of Adhesion Molecules by TNF-*α* in Astrocytes Isolated from WKY/IZM and SHRSP/IZM

**Figure 1:**
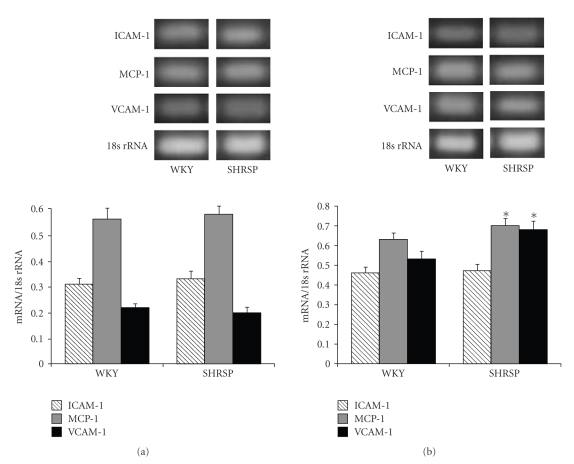
A comparison between the gene expression levels of ICAM-1, MCP-1, and VCAM-1 in astrocytes isolated from WKY/IZM and SHRSP/IZM. The astrocytes isolated from WKY/IZM and SHRSP/IZM were washed with FBS-free DMEM, before TNF-*α* was added (a) or not added (b) at 4 hours. Total cellular RNA was isolated from cultured astrocytes. The RNA was transcribed with reverse transcriptase and amplified by RT-PCR. An 18s ribosomal RNA amplicon was used as an internal control for quantitation of the total amount of cDNA. The bars represent the mean ± SD (*n* = 4). Asterisk (*): *P* < .05 compared with the WKY/IZM value. WKY: WKY/IZM; SHRSP: SHRSP/IZM.

We investigated the expression levels of ICAM-1, MCP-1, and VCAM-1 mRNA in WKY/IZM and SHRSP/IZM astrocytes by exposure to TNF-*α* (10 ng/mL). As shown in Figure 1(a), the expression levels of ICAM-1 and MCP-1 without TNF-*α* were the same in WKY/IZM and SHRSP/IZM at 4 hours. On the other hand, the expression levels of VCAM-1 and MCP-1 mRNA after exposure to TNF-*α* in SHRSP/IZM were significantly (*P *< .05) higher than those in the astrocytes isolated from WKY/IZM (Figure 1(b)).

### 3.2. The TNF-*α*-Induced VCAM-1 Protein Expression Levels of WKY/IZM and SHRSP/IZM Astrocytes and It Inhibition Effect of Apigenin by Western Blotting

**Figure 2:**
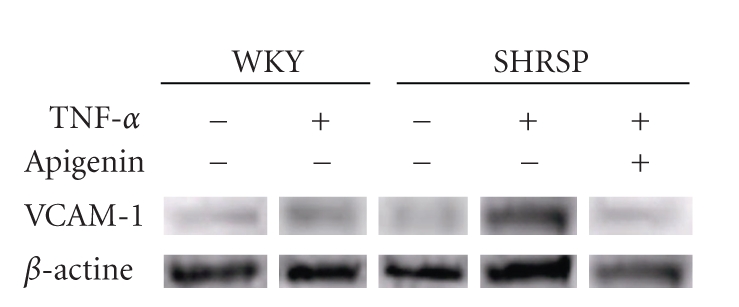
Effects of TNF-*α* and apigenin on VCAM-1 protein expression levels in astrocytes isolated from WKY/IZM and SHRSP/IZM. The astrocytes isolated from WKY/IZM and SHRSP/IZM were incubated with TNF-*α* (10 ng/mL) for 24 hours. Levels of VCAM-1 protein were determined by Western blot analysis.

VCAM-1 activation plays an important role in monocyte adhesion to astrocytes. To confirm the simultaneous protein expression of VCAM-1 we treated the cells with TNF-*α* (10 ng/mL) and apigenin (50 *μ*M) for 24 hours. The astrocytes isolated from SHRSP/ IZM were incubated with TNF-*α* (10 ng/mL) and VCAM-1 protein expression was studied. As shown in Figure 2, the protein expression of VCAM-1 was enhanced by the TNF-*α* treatments. Furthermore, the expression levels of VCAM-1 protein after exposure to TNF-*α* in SHRSP/IZM were significantly (*P *< .05) higher than those in the astrocytes isolated from WKY/IZM. On the other hand, treatment with apigenin clearly reduced VCAM-1 protein expression.

### 3.3. The Effects of Apigenin and Resveratrol on the TNF-*α*-Induced Adhesion Molecule Gene Expression Levels of SHRSP/IZM Astrocytes: Time Courses and Dose Response

**Figure 3:**
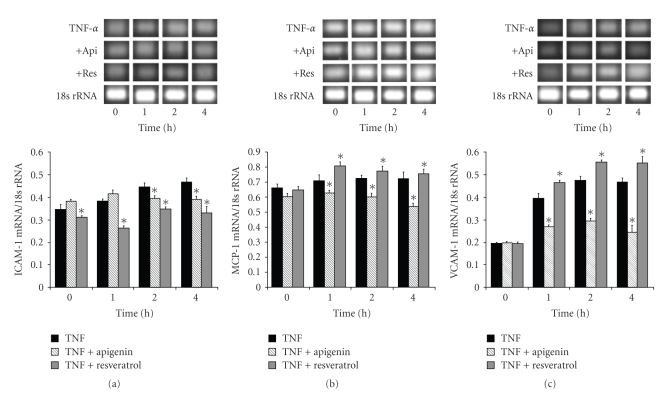
The effects of apigenin and resveratrol on the TNF-*α*-induced expression of ICAM-1, MCP-1, and VCAM-1 in astrocytes isolated from SHRSP/IZM. Time course: the astrocytes isolated from SHRSP/IZM were washed with FBS-free DMEM, before (10 *μ*M) TNF-*α* and apigenin or resveratrol were added. The astrocytes were cultured for 1, 2, or 4 hours. The ICAM-1(a), MCP-1 (b), and VCAM-1 (c) mRNA measurements were carried out as described in Figure 1 and in the text. The bars represent the mean ± SD (*n* = 4). Asterisk (*): *P* < .05 compared with the expression level of TNF-*α* value at the same time. +Api; apigenin, +Res: resveratrol.

**Figure 4:**
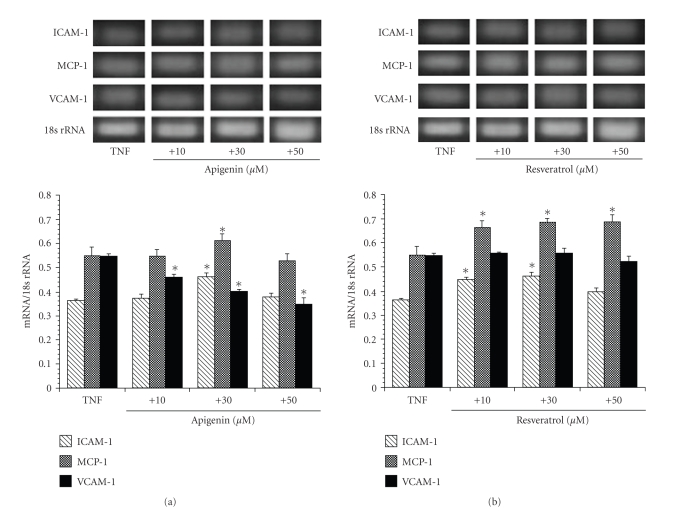
The effects of apigenin and resveratrol on TNF-*α*-induced expression of ICAM-1, MCP-1, and VCAM-1 in astrocytes isolated from SHRSP/IZM. Dose response: the astrocytes isolated from SHRSP/IZM were washed with FBS-free DMEM, replaced with DMEM containing TNF-*α* (10 ng/mL), and supplemented with 0, 10, 30, or 50 *μ*M of apigenin (a) or resveratrol (b). They were then incubated for 4 hours. The ICAM-1, MCP-1, and VCAM-1 mRNA measurements were carried out as described in Figure 1 and in the text. The bars represent the mean ± SD (*n* = 4). Asterisk (*): *P* < .05 compared with the basal TNF-*α* value. TNF: TNF-*α*.

We further examined whether TNF-*α* (10 ng/mL) was able to induce the expression of ICAM-1, MCP-1, or VCAM-1 mRNA in the astrocytes of SHRSP/IZM. Simultaneously, we investigated the inhibitory effects of 10 *μ*M of apigenin or resveratrol on the TNF-*α* induced expression of these molecules over a short period. TNF-*α* (10 ng/mL) was added to astrocytes for 0, 1, 2, or 4 hours to induce the expression of ICAM-1, MCP-1, or VCAM-1 mRNA (Figures 3(a), 3(b), and 3(c)). As shown in Figure 3(a), following the exposure to TNF-*α*, the ICAM-1 mRNA levels increased time-dependently. The level of ICAM-1 mRNA at 4 hours increased 1.35-fold compared with the 0 hour control. Apigenin and resveratrol significantly (*P *  < .05) inhibited TNF-*α* induced ICAM-1 mRNA expression to its basal level. Also, the expression of MCP-1 increased time-dependently after TNF-*α* exposure for 1, 2, or 4 hours (Figure 3(b)). The level of MCP-1 mRNA at 4 hours was slightly increased to 1.10-fold of that of the 0 hours control. Apigenin decreased (*P *< .01) the TNF-*α*-induced expression of MCP-1, but resveratrol increased it at 1, 2, and 4 hours. In addition, the expression levels of VCAM-1 mRNA after exposure to TNF-*α* were strongly increased to 2.01-fold (1 hour), 2.42-fold (2 hour), and 2.38-fold (4 hour) compared with 0 hours control, respectively, (Figure 3(c)). The level of VCAM-1 (2.38-fold) mRNA at 4 hours was higher than those of ICAM-1 (1.35-fold) and MCP-1 (1.10-fold) after treatment with 10 ng/mL TNF-*α*. Apigenin strongly inhibited the augmented expression of VCAM-1 mRNA. On the other hand, resveratrol enhanced the augmented expression of VCAM-1 mRNA. Moreover, we examined the dose-dependent effects of apigenin and resveratrol after exposure to TNF-*α* for 4 hours. As shown in Figure 4(a), apigenin inhibited the dose-dependent expression of ICAM-1 and VCAM-1 mRNA induced by TNF-*α* exposure at 10, 30, and 50 *μ*M but did not do the same for MCP-1. On the other hand, resveratrol enhanced the expression levels of MCP-1 mRNA induced by TNF-*α* exposure at 10, 30, and 50 *μ*M. These adhesion molecule gene expression patterns induced by TNF-*α* exposure were dose-dependent over the course of the short examination period. Concomitantly, we pointed out the inhibitory effects of apigenin on the gene expression patterns of adhesion molecules.

### 3.4. A Comparison between the Gene Expression Levels of Adhesion Molecules during Hypoxia and H/R in Astrocytes Isolated from WKY/IZM and SHRSP/IZM

**Figure 5:**
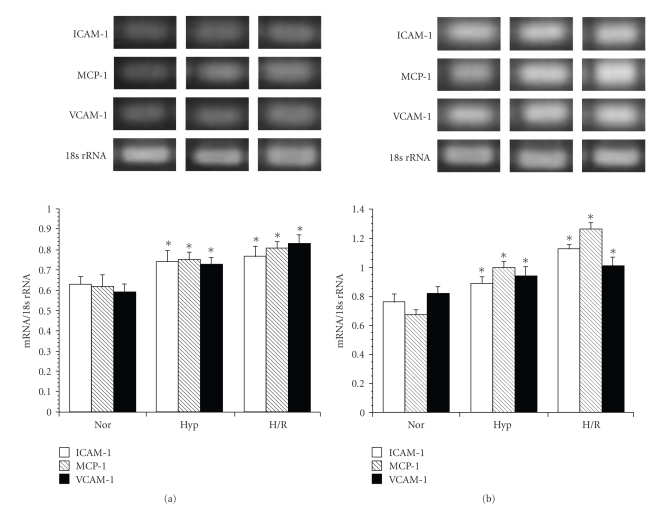
A comparison between the gene expression levels of ICAM-1, MCP-1, and VCAM-1 in astrocytes isolated from WKY/IZM and SHRSP/IZM during hypoxia and reoxygenation. The astrocytes isolated from WKY/IZM (a) or SHRSP/IZM (b) were incubated under hypoxia (1% O2, 24 hour) or hypoxia + reoxygenation (21% O2, 3 hour) as previously described [13, 15]. The ICAM-1, MCP-1, and VCAM-1 mRNA measurements were carried out as described in Figure 1 and in the text. The bars represent the mean ± SD (*n* = 4). Asterisk (*): *P* < .05 compared with the normal condition values. Nor: normal condition, Hyp: Hypoxia, H/R: hypoxia and reoxygenation.

We investigated the expression levels of ICAM-1, MCP-1, and VCAM-1 mRNA in WKY/IZM and SHRSP/IZM astrocytes during normoxia, hypoxia, and H/R. As shown in Figure 5, in both WKY/IZM (Figure 5(a)) and SHRSP/IZM (Figure 5 (b)) hypoxia and H/R induced the expression of ICAM-1, MCP-1, and VCAM-1 mRNA compared with normoxic conditions. The expression levels of ICAM-1, MCP-1, and VCAM-1 mRNA in the WKY/IZM astrocytes were similar during hypoxia and H/R. On the other hand, the expression of ICAM-1, MCP-1, and VCAM-1 of SHRSP/IZM were higher than those of WKY/IZM during hypoxia and H/R, respectively, and the expression levels of ICAM-1, MCP-1, and VCAM-1 in the SHRSP/IZM were higher than those in WKY/IZM during hypoxia and H/R, respectively. Furthermore, the expression of MCP-1 mRNA during H/R in the SHRSP/IZM astrocytes was markedly increased.

### 3.5. The Effects of Extending the Exposure Period of the Astrocytes to Apigenin or Resveratrol before and/or after Hypoxia

**Figure 6:**
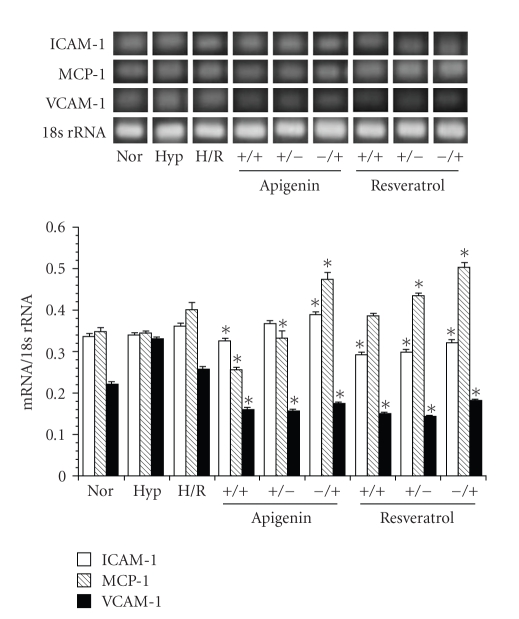
The effects of H/R on the expression levels of adhesion molecules in astrocytes isolated from SHRSP and a comparison between the inhibitory effects of apigenin, resveratrol. The astrocytes isolated from SHRSP/IZM was incubated under hypoxia (1% O2, 24 hour) or hypoxia + reoxygenation (21% O2, 3 hour) as previously described [13, 15]. They were incubated for an additional period either before hypoxia or after hypoxia with 50 *μ*M of apigenin or resveratrol. The ICAM-1, MCP-1, and VCAM-1 mRNA measurements were carried out as described in Figure 1 and in the text. The bars represent the mean ± SD (*n* = 4). Asterisk (*): *P* < .05 compared with the normal condition values. Nor: normal condition; Hyp: Hypoxia; H/R: hypoxia and reoxygenation.

Furthermore, we examined the effects of extending the exposure period of the astrocytes to apigenin or resveratrol before hypoxia and/or after hypoxia on adhesion molecule expression in SHRSP/IZM astrocytes. The astrocytes were incubated for a longer period before hypoxia or after hypoxia, with 10 *μ*M of apigenin or resveratrol. As shown in Figure 6, the expression of adhesion molecules was inhibited when the length of the exposure period to apigenin or resveratrol was extended both before and after hypoxia. However, the expression levels of the adhesion molecules were enhanced when apigenin or resveratrol was added for longer periods before or after hypoxia.

### 3.6. The Effects of Apigenin, Resveratrol, Ebselen, NAC, and Pioglitazone on H/R-Induced Adhesion Molecule Gene Expression Levels in SHRSP/IZM Astrocytes

**Figure 7:**
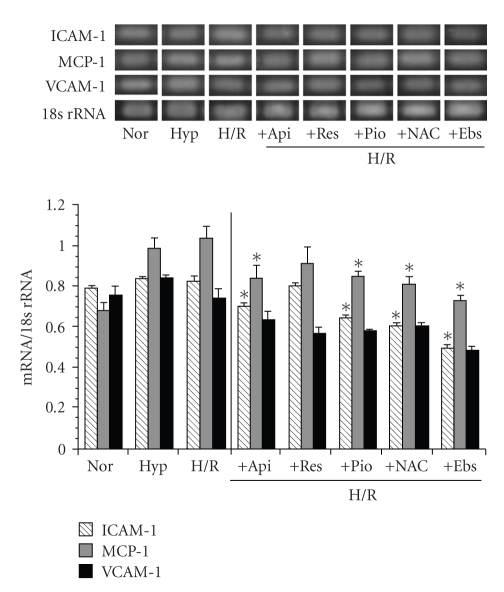
The effects of H/R on the expression levels of adhesion molecules in astrocytes isolated from SHRSP/IZM and a comparison of the inhibitory effects of apigenin and resveratrol, ebselen, NAC, and pioglitazone. The astrocytes isolated from SHRSP/IZM were incubated under hypoxia (1% O2, 24 hour) or hypoxia + reoxygenation (21% O2, 3 hour) as previously described [13, 15]. They were incubated for an additional period either before or after the hypoxia with 50 *μ*M of apigenin, resveratrol, ebselen, NAC, or pioglitazone. The ICAM-1, MCP-1, and VCAM-1 mRNA measurements were carried out as described in Figure 1 and in the text. The bars represent the mean ± SD (*n* = 4). Asterisk (*): *P* < .05 compared with the normal condition values. Nor: normal conditions; Hyp: Hypoxia; H/R: hypoxia and reoxygenation.

We investigated the effects of apigenin, resveratrol, ebselen, NAC, and pioglitazone on the H/R-induced adhesion molecule expression levels of SHRSP/IZM astrocytes. All agents were added before hypoxia and reoxygenation. As shown in Figure 7, we indicated the H/R-induced expression levels of adhesion molecules and the inhibitory effects of apigenin, resveratrol, pioglitazone, NAC, and ebselen. Apigenin, resveratrol, ebselen, NAC, and pioglitazone all reduced the expression levels of MCP-1 and VCAM-1 mRNA. In particular, ebselen strongly decreased the H/R treated expression levels of ICAM-1, MCP-1, and VCAM-1. The strength of the inhibition of the expression of adhesion molecules was in the following order: ebselen > NAC, apigenin = pioglitazone > resveratrol.

### 3.7. The Inhibitory Effects of Apigenin on U937 Cell Adhesion in Cultured SHRSP/IZM Astrocytes

**Figure 8:**
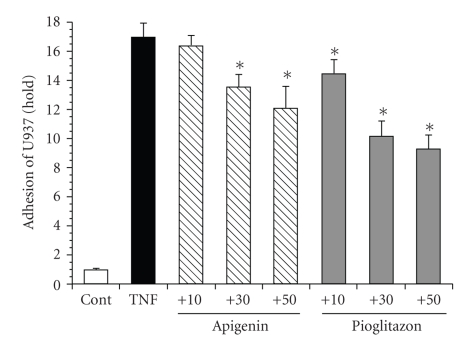
The inhibitory effects of apigenin and pioglitazone on U937 cell adhesion in astrocytes isolated from SHRSP. The astrocytes isolated from SHRSP/IZM were cultured for 24 hours with or without 10, 30, or 50 *μ*M of apigenin or pioglitazone and were then challenged with TNF-*α* (10 ng/mL). The U937 cells (1 × 10^ 6^ cells/mL) were added and incubated with astrocytes at 37°C for 3 hours. The adhesion of U937 cells to the astrocytes was measured using quantitative monolayer adhesion assays, similar to those previously described [28]. The inhibitory effects of apigenin are indicated (fold). The number of bound U937 cells per square millimeter was determined by direct microscopy. The data are means ± S.D (*n* = 6).

The adhesion of U937 cells to astrocytes was measured using quantitative monolayer adhesion assays [28]. Pretreatment of primary SHRSP/IZM astrocyte monolayers for 24 hours with 10 ng/mL of TNF-*α* resulted in an increase in the adhesion of U937 cells (Figure 8). Under similar conditions, 30 *μ*M and 50 *μ*M of apigenin inhibited the adhesion of U937 cells. In addition, pioglitazone strongly inhibited the adhesion of U937 cells at 10 *μ*M, 30 *μ*M, and 50 *μ*M. The inhibitory effect of pioglitazone was concentration dependent. Apigenin inhibited the adhesion of U937 cells; however, the effect of apigenin was weak compared with that of pioglitazone.

## 4. Discussion

The expression of adhesion molecules in astrocytes after H/R has been implicated in the pathogenesis of SHRSP [16]. In this study, we showed that the expression of VCAM-1 mRNA and VCAM-1 protein induced by TNF-*α* in astrocytes isolated from SHRSP/IZM is higher than that induced in WKY/IZM. Furthermore, we examined the H/R-induced expression levels of MCP-1 in SHRSP/IZM astrocytes. Apigenin reduced the expression levels of ICAM-1, MCP-1, and VCAM-1. In particular, treatment with apigenin clearly reduced the VCAM-1 gene and VCAM-1 protein expression.

 Leukocyte infiltration into the brain has been implicated in the development of ischemic brain damage. A previous study demonstrated that in vitro ischemia/reperfusion and IL-1 *β* upregulate the expression of ICAM-1 in cultured human endothelial cells and human fetal astrocytes [30]. Astrocytes respond to IL-1 *β* or in vitro ischemia/reperfusion with a pronounced upregulation of IL-8 and MCP-1 mRNA and by increased release of IL-8 and MCP-1 in cell culture media. Astrocytes were found to release much higher levels of MCP-1 than endothelial cells under basal and ischemic conditions. Furthermore, a previous report demonstrated that RANTES (regulated on activation, normal T-cell expressed and secreted), which is also known as CCL5, induces TNF-*α*, which in turn stimulates the production of MCP-1. On the other hand, a central role for astrocyte-derived MCP-1 in leading the migration of monocytes and lymphocytes across the blood-brain barrier (BBB) has been indicated [31]. Consequently, astrocytes are considered as proinflammatory cascade amplified and synthesize proinflammatory mediators [32]. In SHRSP/IZM, the expression levels of MCP-1 are dramatically increased two days after ischemia-reperfusion. It has been suggested that the onset of stroke is related to the rapid increase in MCP-1 due to ischemia-reperfusion in SHRSP [16]. We therefore demonstrated that the expression levels of VCAM-1 increase after TNF-*α* treatment in astrocytes derived from SHRSP/IZM and showed that MCP-1 expression increases during H/R. These findings support the assertion that H/R induces the expression of MCP-1, increases leukocytic infiltration, and promotes leukocyte adhesion to brain cells in SHRSP/IZM.

Next, we examined the inhibitory effects of the dietary flavonoids apigenin and resveratrol on the expression levels of IVAM-1, VCAM-1, and MCP-1 after TNF-*α* treatment. Apigenin inhibited the TNF-*α* induced expression levels of ICAM-1, MCP-1, and VCAM-1. On the other hand, resveratrol reduced the expression levels of VCAM-1 but did not reduce the expression levels of ICAM-1 or MCP-1. Furthermore, we demonstrated that apigenin as well as pioglitazone attenuates the H/R-induced expression of MCP-1 mRNA. A recent study showed that apigenin inhibits the secretion of nitric oxide (NO) and prostaglandin E_2_ by suppressing the expression of inducible nitric oxide synthase (NOS) and cyclooxygenase-2 (COX-2) in murine microglia cell lines. In addition, apigenin was also shown to protect neuronal cells from injury after middle cerebral artery occlusion (MCAO). Although it has been suggested that astrocyte activation in SHRSP/IZM may be implicated in the pathogenesis of stroke [16], the inhibitory effects of flavonoids including apigenin on the expression patterns of SHRSP/IZM astrocytes have not yet been elucidated. In this study, we demonstrated that apigenin inhibits the expression of several adhesion molecules in SHRSP/IZM. Ebselen (2-phenyl-1,2-benzisoselenazol-3[2H]-one), a selenoorganic compound, is effective for treating acute ischemic stroke; however, its effect on SHRSP/IZM has not yet been elucidated. We have previously demonstrated that ebselen is effective for the prevention and/or treatment of neurodegenerative diseases associated with excessive apoptosis in patients with stroke [15]. In this study, ebselen reduced the expression levels of ICAM-1 and VCAM-1 mRNA. Other reports have demonstrated that TNF-*α*-induced c-Jun N-terminal kinase (JNK) activation is inhibited by ebselen in cultured human umbilical vein endothelial cells (HUVECs). In addition, the reduction of JNK by ebselen implied its usefulness for the prevention of atherosclerosis, which is related to endothelial cell activation [33]. It has been suggested that the protective efficacy of ebselen against neurodegenerative diseases might be implicated in the inhibitory effects of adhesion molecule expression, the inhibition of JNK [33], antioxidant effects [34], and the actions of glutathione mimics [35]. Also, we have demonstrated that pioglitazone inhibits the expression of MCP-1 mRNA. Pioglitazone is associated with a lower risk of death, myocardial infarction, and stroke in patients with diabetes [36]. Furthermore, pioglitazone shows protective effects against stroke in SHRSP/IZM, independently of blood pressure [37]. The peroxisome proliferator-activated receptor- (PPAR-) gamma agonist pioglitazone reduces the incidence of stroke in patients with type 2 diabetes. These actions of pioglitazone in astrocytes of SHRSP/IZM may occur through stimulation of PPAR-gamma [23]. The potent antioxidant NAC has been demonstrated to attenuate cerebral ischemia-reperfusion injury in a rat model of experimental stroke [38]. NAC reduced the expression of TNF-*α*, IL-1 beta, and iNOS. Furthermore, a previous report demonstrated that NAC, an antioxidant that inhibits NF-kappa B activation, alters events in brain reperfusion injury. NAC attenuates cerebral infarction by blocking activation of NF-kappa B [39]. Furthermore, another report indicated that apigenin inhibits p38 mitogen-activated protein kinase (MAPK) JNK phosphorylation without affecting the activity of extracellular signal-regulated kinase (ERK) [40]. In addition, the inhibitory effects of apigenin on ICAM-1 expression are mediated by the attenuation of the several MAPK activities, c-fos and c-jun mRNA expression, and AP-1 transcriptional activity and inhibit NF-kappa B [41, 42]. However, apigenin may decrease the expression levels of adhesion molecules such as MCP-1 and VCAM-1 by blocking the activation of NF-kappa B in SHRSP/IZM during H/R [38, 41].

## 5. Conclusions

In conclusion, we have demonstrated the TNF-*α* and H/R-induced expression of adhesion molecules in primary astrocytes of WKY/IZM and/or SHRSP/IZM. Furthermore, apigenin, resveratrol, ebselen, pioglitazone, and NAC were shown to modify adhesion molecule expression. Concomitantly, apigenin and pioglitazone strongly reduced the adhesion of primary astrocytes isolated from SHRSP/IZM and U937. These findings suggest that the expression of adhesion molecules in astrocytes after H/R is implicated in the pathogenesis of SHRSP/IZM. Moreover, it is suggested that apigenin regulates the expression of adhesion molecules in reactive astrocytes during ischemic statuses such as stroke.

## Figures and Tables

**Table 1:** Sequences of primas for RT-PCR.

Gene	Forward primer (5′ → 3′)	Reverse primer (5′ → 3′)	Amplicon (bp)	Accession number
ICAM-1	TGTCAAACGGGAGATGAATGGT	CGTCCCTGGTGATACTCCCA	71	NM_012967
MCP-1	GATGCAGTTAATGCCCCACTC	CCAGCCGACTCATTGGGA	70	NM_031530
VCAM-1	GCTCGTACACCATCCGCAAG	CGGTTTTCGATTCACACTCGT	66	NM_012889

## Abbreviations

H/R: Hypoxia and reoxygenation
ICAM-1: Intercellular adhesion molecule-1
IL-6: Interleukin-6
MCP-1: Monocyte chemotactic protein-1
MCAO: Middle cerebral artery occlusion
NAC: N-acetylcysteine
SHRSP/IZM: Stroke-prone spontaneously hypertensive rats
TNF-*α*: Tumor necrosis factor-alpha
VCAM-1: Vascular cell adhesion molecule-1
WKY/IZM: Wistar Kyoto rats.
